# Four-factor prothrombin complex concentrate to reduce allogenic blood product transfusion in patients with major trauma, the PROCOAG trial: study protocol for a randomized multicenter double-blind superiority study

**DOI:** 10.1186/s13063-021-05524-x

**Published:** 2021-09-16

**Authors:** Pierre Bouzat, Jean-Luc Bosson, Jean-Stéphane David, Bruno Riou, Jacques Duranteau, Jean-François Payen, on behalf of the PROCOAG study group, Albrice Levrat, Paër-Selim Abback, Jacques Duranteau, Bruno Riou, Delphine Garrigue, Guillaume Marcotte, Jean-Stéphane David, Jonathan Charbit, Karim Asehnoune, Marc Leone, Julien Pottecher, Pierre Bouzat

**Affiliations:** 1 Pôle Anesthésie-Réanimation, Centre Hospitalo-Universitaire Grenoble-Alpes, Grenoble, France; 2 Univ. Grenoble Alpes, CNRS, Public Health department CHU Grenoble Alpes, TIMC-IMAG, 38000 Grenoble, France; 3 Service d’Anesthésie Réanimation, Centre Hospitalo-Universitaire Lyon-Sud, Faculté de Médecine Lyon-Est, Université Claude Bernard Lyon 1, Lyon, France; 4 Sorbonne Université, UMRS INSERM 116, IHU ICAN, et Service des urgences, Hôpital Pitié-Salpêtrière, Assistance Publique-Hôpitaux de Paris, Paris, France; 5 Département d’Anesthésie-Réanimation, Hôpitaux Universitaires Paris Sud, Université Paris XI, Faculté de Médecine Paris-Sud, Le Kremlin-Bicêtre, France

**Keywords:** Acute traumatic coagulopathy, Prothrombin complex concentrate, Severe trauma, Massive transfusion

## Abstract

**Background:**

Optimal management of severe trauma patients with active hemorrhage relies on adequate initial resuscitation. Early administration of coagulation factors improves post-traumatic coagulation disorders, and four-factor prothrombin complex concentrate (PCC) might be useful in this context. Our main hypothesis is that four-factor PCC in addition to a massive transfusion protocol decreases blood product consumption at day 1 in severe trauma patients with major bleeding.

**Methods:**

This is a prospective, randomized, multicenter, double-blind, parallel, controlled superiority trial. Eligible patients are trauma patients with major bleeding admitted to a French level-I trauma center. Patients randomized in the treatment arm receive 1 mL/kg (25 IU/ml of Factor IX/Kg) four-factor PCC within 1-h post-admission while patients randomized in the controlled group receive 1 mL/kg of saline solution 0.9% as a placebo. Treatments are given as soon as possible using syringe pumps (120 mL/h). The primary endpoint is the amount of blood products transfused in the first 24 h post-admission (including red blood cells, frozen fresh plasma, and platelets). The secondary endpoints are the amount of each blood product transfused in the first 24 h, time to achieve prothrombin time ratio < 1.5, time to hemostasis, number of thrombo-embolic events at 28 days, mortality at 24 h and 28 days, number of intensive care unit-free days, number of ventilator-free days, number of hospital-free days within the first 28 days, hospitalization status at day 28, Glasgow outcome scale extended for patients with brain lesions on initial cerebral imaging, and cost of each strategy at days 8 and 28. Inclusions have started in December 2017 and are expected to be complete by June 2021.

**Discussion:**

If PCC reduces total blood consumption at day 1 after severe trauma, this therapy, in adjunction to a classic massive transfusion protocol, may be used empirically on admission in patients at risk of massive transfusion to enhance coagulation. Moreover, this treatment may decrease blood product-related complications and may improve clinical outcomes after post-traumatic hemorrhage.

**Trial registration:**

ClinicalTrials.gov NCT03218722. Registered on July 14, 2017

## Administrative information

The order of the items has been modified to group similar items (see http://www.equator-network.org/reporting-guidelines/spirit-2013-statement-defining-standard-protocol-items-for-clinical-trials/).

**Table:** 

Title {1}	Four-factor prothrombin complex concentrate to reduce allogenic blood product transfusion in patients with major trauma, the PROCOAG trial: study protocol for a randomized multicenter double-blind superiority study.
Trial registration {2a and 2b}.	Clinical trial, NCT03218722
Protocol version {3}	Date: February 6^th^ 2019, Version: 6.0
Funding {4}	PROCOAG is an investigator-initiated trial, supported by a non-profit grant from the French Ministry of Health (PHRCI 2015, n°15-059). LFB (Les Ullis, France) is the manufacturer of the pharmaceutical product used in the trial and distributes Kanokad® for free to participating centers.
Author details {5a}	Pr. Pierre Bouzat and Pr. Jean-François Payen : Pôle Anesthésie-Réanimation, Centre Hospitalo-Universitaire Grenoble-Alpes, Grenoble, France Pr. Jean-Luc Bosson : Univ. Grenoble Alpes, CNRS, Public Health department CHU Grenoble Alpes, TIMC-IMAG, 38000 Grenoble, France Pr. Jean-Stéphane David : Service d’Anesthésie Réanimation, Centre Hospitalo-Universitaire Lyon-Sud, Faculté de Médecine Lyon-Est, Université Claude Bernard Lyon 1, Lyon, France Pr. Bruno Riou : Sorbonne Université, UMRS INSERM 116, IHU ICAN, et Service des urgences, Hôpital Pitié-Salpêtrière, Assistance Publique-Hôpitaux de Paris, Paris, France Pr. Jacques Duranteau : Département d’Anesthésie-Réanimation, Hôpitaux Universitaires Paris Sud, Université Paris XI, Faculté de Médecine Paris-Sud, Le Kremlin-Bicêtre, France.
Name and contact information for the trial sponsor {5b}	French Ministry of Health, GIRCI AURA, 3 Quai des Célestins, 69002 Lyon, France. PHRCI 2015, n°15-059
Role of sponsor {5c}	Funders have no role in trial design, conduction, data collection, nor analysis. Kanokad® safety aggregated data are shared with LFB.

## Introduction

### Background and rationale {6a}

Severe trauma is the leading cause of mortality in young adults in developed countries and is the third cause of overall death in the USA [1]. Death occurs within the first 48 h among 87% of trauma patients with fatal outcome, and the first cause of death at 24 h is massive bleeding [2, 3]. Hemorrhagic shock is preventable with adequate strategies including early control of the active source of bleeding and an optimization of initial resuscitation called “damage control resuscitation” [4]. The concept of damage control resuscitation consists of a combination of early administration of blood products, control of post-traumatic coagulopathy, and a reduction of crystalloid infusion and maintaining permissive hypotension. This strategy aims at treating hypovolemia and coagulation disorders while avoiding dilution coagulopathy [4]. International guidelines for blood product administration recommend the transfusion of packed red blood cell (RBC) and fresh frozen plasma (FFP) as soon as possible with RBC/FFP ratio between 1:1 and 2:1 [5, 6].

Early administration of coagulation factors with FFP has been highlighted in the prehospital setting suggesting a potential beneficial effect on mortality [7]. However, FFP administration may be delayed due to logistical reasons such as transport and immediate availability. The administration of four-factor prothrombin complex concentrate (PCC) has emerged as an alternative to early administrate coagulation factors and is used to treat post-traumatic coagulopathy in diverse European countries [8, 9]. A strategy combining fibrinogen and four factor-PCC has decreased blood product consumption [8]. The reduction of blood product consumption is meaningful since blood transfusion is associated with post-traumatic infectious complications [10] and is a risk factor for multiple organ failure after severe trauma [11]. Recently, an open-label randomized controlled trial comparing a classic massive transfusion protocol to a tailored administration of procoagulant concentrates, including PCC and fibrinogen, also showed a decrease in RBC transfusion resulting in less patients requiring massive transfusion [12]. However, there is no randomized controlled double-blind study assessing the impact of PCC in addition to a classic massive transfusion protocol including fibrinogen administration and blood product transfusion with RBC and FFP [13].

The PROCOAG study therefore aims to test whether early administration of PCC allows a reduction of the total number of blood products transfused within the first 24 h after hospital admission of trauma patients with major hemorrhage.

### Objectives {7}

The primary objective of the PROCOAG study is to test whether the early PCC administration with standard blood products compared with standard care alone allows better control of post-traumatic hemorrhage, echoed by the total number of blood products transfused within the first 24 h after hospital admission.

Secondary objectives are as follows:

- To conduct a separate analysis of the primary outcome:
  - Assessment of RBC consumption
  - Evaluation of FFP consumption
  - Evaluation of platelet consumption
- To compare between the two groups:
  - Time to normalize hemostasis defined by the time to achieve prothrombin time ratio < 1.5
  - Time from admission to hemorrhage control defined during a surgical and/or radiological procedure, defined as the time necessary to control bleeding in the surgical field (operative field is dry without further foreseeable homeostatic procedures) or to control contrast blush after embolization during an interventional radiology (no more dye flushing).
  - Thromboembolic events (pulmonary embolism, deep venous thrombosis)
  - Morbi-mortality: mortality at 24 h and 28 days, intensive care unit (ICU)-free days (number of in-hospital days outside ICU) within the first 28 days, ventilator-free days during ICU stay within the first 28 days, hospital-free days within the first 28 days, and hospitalization status at day 28,
  - Neurological outcome (Glasgow Outcome Scale Extended, GOS-E) for patients with brain lesions on initial scan
- To evaluate differences in terms of cost-effectiveness

### Trial design {8}

The PROCOAG study is a therapeutic, prospective, randomized, multicenter, double-blind, parallel, superiority controlled clinical trial testing the efficacy of four factor-PCC to reduce the amount of blood products transfused in patients at risk of massive transfusion.

## Methods: participants, interventions, and outcomes

### Study setting {9}

Twelve French academic hospitals will participate in the study. All centers are level-I trauma centers receiving more than 200 trauma patients with an injury severity score (ISS)>15 each year. A list of all participating centers can be found on https://clinicaltrials.gov.

### Eligibility criteria {10}

#### Inclusion criteria

All consecutively admitted adult patients at risk of massive transfusion after severe trauma are screened for enrolment. Patients older than 18 years, admitted directly from the injury scene to a level-I trauma center, are eligible provided that they have two criteria: (1) a transfusion of at least one unit (U) of RBC prior to hospital admission or within the first hour after their admission (a delay of 90 min is tolerated if transfusion is decided within 1 h post admission) and (2) prediction by an assessment of blood consumption (ABC) score ≥ 2 [14] or by physician judgment of the need for a massive transfusion (defined by a transfusion of at least 10 RBC during the first 24 h or 3 RBC during the first hour).

#### Exclusion criteria

Patients are not eligible in the case of (1) traumatic cardiac arrest before randomization, (2) secondary admission from another health care facility/hospital (technical shortstops are accepted, i.e., such a stop should not 1 h before admission to the trauma center), (3) treatment with anticoagulants, (4) known pregnancy, (5) known hypersensitivity to four-factor PCC or its excipients, (6) patient with devastating injuries expected to die within the first hour post-admission, (7) patient under therapeutic limitation before randomization, (8) patient under guardianship, (9) knowledge of a contraindication to the use of NaCl 0.9% at the dose of 1 mL/kg (e.g., hyperchloremia or hypernatremia), or a drop-out criterion (10) inclusion in another trial within the last 30 days, (11) family or patient refusal, and (12) patient without health insurance.

### Who will take informed consent {26a}

Considering the study context, a considerable number of enrolled patients will be unable to consent (e.g., unconsciousness, agitation, post-traumatic stress). Informed consent can be therefore obtained from patient’s next of kin if present or consent is waived if no one is present at the time of enrolment, as authorized by French law in an emergency situation and approved by the ethics committee. This procedure was already used and authorized in a previous French study on trauma [15]. As soon as the patient’s next of kin is available and capable of informed consent in complete and faithful terms, consent is obtained. Informed consent must also be obtained from the patient whenever he/she becomes able to consent and up to 28 days after trauma. Patients and their next of kin have the right to refuse participation in the study or revoke their consent at any time. All information appears in an information notice and a consent form given to the subject. All these documents have been approved by the ethics committee. Written informed consent is obtained by the investigator. Two original copies are co-signed by both the investigator and the subject who consent.

### Additional consent provisions for collection and use of participant data and biological specimens {26b}

This is not applicable.

### Interventions

#### Explanation for the choice of comparators {6b}

The comparator will be normal saline (NaCl 0.9%, dose: 1ml/kg). At this dose, NaCl is neutral regarding its interaction with the coagulation factors and therefore considered a placebo.

#### Intervention description {11a}

Within 1 h of admission, patients are randomized in two arms. In the placebo arm, patients are managed following standard care in addition to intravenous 1ml/kg saline solution. In the treatment arm, patients are managed following standard care in addition to intravenous administration of 1ml/kg four-factor PCC (25 IU/ml of Factor IX/kg). Treatments are given as soon as possible using syringe pumps (120 mL/h).

All centers follow European guidelines for the management of post-traumatic hemorrhage. Details of standard management are found below (11d).

#### Criteria for discontinuing or modifying allocated interventions {11b}

Study treatment is managed by the investigating team, starting within 1-h post-admission and is administered in intravenous perfusion at 2mL per minute. There is no reason for discontinuing or modifying treatment unless an adverse reaction to the study treatment occurs. In this unlikely event, perfusion should be stopped, appropriate care provided immediately, and the event should be reported to the sponsor. Dose change is not permitted by the protocol.

#### Strategies to improve adherence to interventions {11c}

Because the investigating team administrates the treatment, no strategy is needed to improve patient adherence to the intervention.

#### Relevant concomitant care permitted or prohibited during the trial {11d}

From admission, all patients are managed according to European recommendations. Briefly, the source of bleeding should be identified as soon as possible and treated with emergency surgery and/or embolization following standard practice [16]. Regarding resuscitation, patients are treated in both groups with restricted volume replacement strategy, early transfusion of blood products with a RBC:FFP ratio between 1:1 and 2:1. Tranexamic acid (TXA) is intravenously administered within 3h after injury at a loading dose of 1 g infused over 10 min followed by an intravenous infusion of 1 g over 8 h [17]. Fibrinogen concentrate is administered in case of fibrinogen concentration <1.5 g/L [18] or viscoelastic evidence of a functional fibrinogen deficiency [19]. Platelets are administered to maintain a platelet count above 50 x 10^9^/L. Vasopressors can be infused in addition to fluids to maintain target systolic arterial blood pressure (>90 mmHg or >110 mmHg for head-injured patients). Blood samples are regularly drawn from admission to test hemostasis (prothrombin time ratio, Quick time, fibrinogen concentration, and viscoelastic tests where available), blood electrolytes, hepatic and renal function, and blood gases.

#### Provisions for post-trial care {30}

Post-trial care is not planned. Patients who suffer harm from trial participation will be cared for in the intensive care unit. Should prejudice linked to study participation occur, financial compensation will be provided by the insurance (Société Hospitalière d’Assurances Mutuelles—SHAM, 18 rue Edouard Rochet, 69,372 Lyon Cedex 08, France) contracted by the promotor.

#### Outcomes {12}

The primary outcome is the total number of blood products transfused within the first 24 h after hospital admission, including RBC, FFP, and platelets. The choice of this primary outcome is based on the following rationale. First, a recent randomized control trial exploring the effect of vasopressin use after severe trauma on the same endpoint [20], Second, even if transfusion is considered a pillar in the management of acute bleeding, blood products are associated with adverse events and poor clinical outcome [11, 21]. Third, mortality after severe trauma is a complex process depending on hemorrhage but also on traumatic brain injury and pre-existing medical conditions, specifically in elderly patients [22]. We thus believe that 24h total blood consumption is an accurate proxy of the severity of the bleeding until the hemorrhage stops.

Secondary outcome measures are (1) number of separate units (and volume) of RBC, FFP, and platelets and transfused in the first 24 h; (2) time to achieve prothrombin time ratio < 1.5; (3) time to hemostasis, defined as the time necessary to control bleeding in the surgical field (operative field is dry without further foreseeable homeostatic procedures) or to control contrast blush after embolization during an interventional radiology (no more dye flushing); (4) number of thrombo-embolic events at 28 days; (5) mortality at 24 h and 28 days; (6) intensive care unit (ICU)-free days (number of in-hospital days outside ICU) within the first 28 days; (7) ventilator-free days during ICU stay within the first 28 days; (8) hospital-free days within the first 28 days; (9) hospitalization status at day 28; (10) GOSE for patients with brain lesions on initial scan; and (11) cost of each strategy at days 8 and 28. Cost analyses will be performed on expensive care: blood products and procoagulant treatments administered, imaging exams, surgeries, and length of stay in each healthcare unit.

#### Participant timeline {13}

**Fig. 1:**
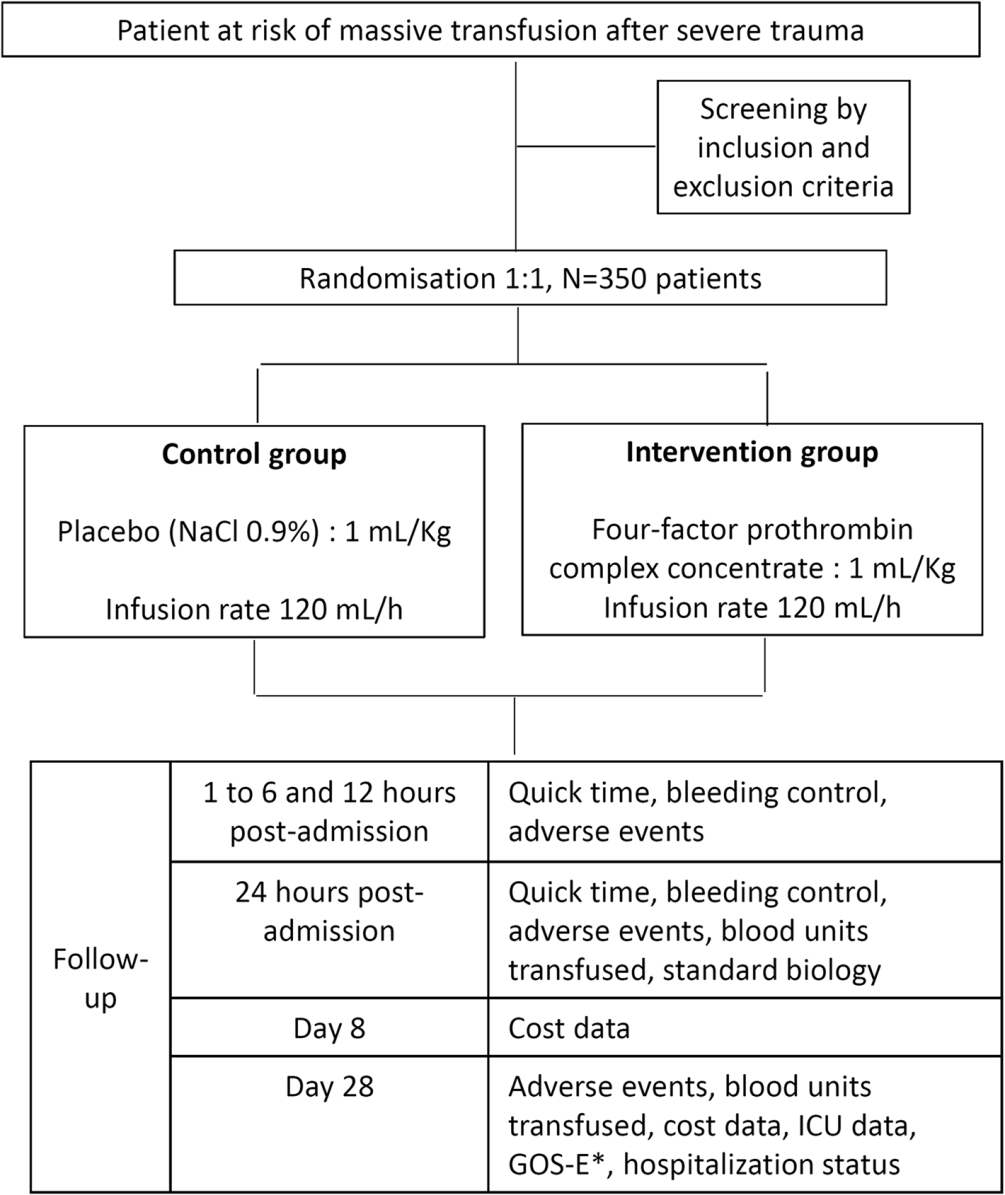
Design of the study

All consecutive trauma patients admitted directly from the injury scene who require RBC transfusion with a risk of massive transfusion are screened on admission. Eligible patients are then rapidly enrolled by the study investigator. They should be randomized and treated accordingly within 60 min post-admission. After patient treatment, follow-up includes hourly visits until 6 h post-admission, as well as visits at 12 and 24 h to measure prothrombin ratio, assess hemorrhage control and monitor adverse events. Thrombo-embolic events are under special scrutiny during the entire hospital stay because they are frequently expected adverse effects of PCC treatment. Each suspicious thrombo-embolic event should be confirmed with venous Doppler echography or angiography and treated following standard practice. Thromboprophylaxis is left to physician discretion. Patients are followed for 28 days with the last visit aiming at collecting the Glasgow outcome scale extended (GOSE) for patients with brain lesions on initial cerebral imaging, mortality, and place of living (Fig. 1).

#### Sample size {14}

Total blood consumption was estimated in the control group based on 1-year consumption in Grenoble University Hospital and Annecy General Hospital; we found that patients with our eligibility criteria received 12 units on average of RBC, FFP, and platelets. We consider a reduction of 3 units as clinically significant since 3 units represent 1 L of blood products. Thus, a 25% decrease in transfused blood products in the first 24 h is expected (from an average of 12 transfused units in the control group to 9 transfused units in the experimental group). The study needs to include 162 patients per group to demonstrate this 25% reduction with 80% power and α-risk of 0.05, bilateral test (nQuery, Sample Size and Power Calculation, “Statsols”, Statistical Solutions Ltd., Cork, Ireland, blood product units transfused do not follow a normal distribution). To compensate for the estimated lost-to-follow-up, the trial plans to include 350 patients.

#### Recruitment {15}

All centers are level-I trauma centers in France with sufficient recruitment of severe trauma patients. Before the start of the study, a feasibility questionnaire has circulated between all centers and they were asked for potential inclusions in the study. They all agreed to include between one and two patients each month. Regular newsletters will be sent and all participating centers are part of a national network for trauma care (GITE group).

### Assignment of interventions: allocation

#### Sequence generation {16a}

Randomization will be performed by sealed envelopes available at the investigator site. Randomization will be by block of random size from 2 to 6, stratified by center. The randomization list will be created and maintained by the study sponsor. Randomization envelopes to be opened will be created prior to the study by the coordinating pharmacy and made available during the site visit. The integrity and presence of the envelopes will be checked at each monitoring visit.

#### Concealment mechanism {16b}

Allocation sequence is implemented through sequentially numbered, opaque, sealed, envelopes. Envelopes have been created by the coordinating pharmacy in Grenoble and provided to each investigating site pharmacy which manages the stock. Site pharmacy provides 4 sequentially numbered envelopes to the investigating team and renew the stock after each randomization. Envelopes are sealed with inviolable tapes making any opening visible. Therefore, the investigating team cannot modify the inclusion decision after allocation without such a modification being noticed by the hospital pharmacy and monitoring team.

#### Implementation {16c}

An independent statistician will generate the allocation sequence, a clinician investigator (clinician trained in trauma resuscitation) will enroll participants; an unblinded, trained nurse not involved in patient care assign participants to one of the study arms by unsealing randomization envelope.

### Assignment of interventions: blinding

#### Who will be blinded {17a}

After enrolment, the blinded clinician investigator conveys to the unblinded, dedicated, trained nurse patient’s name, weight, and study number. This nurse does not participate in the resuscitation of the patient. The nurse prepares the solution to be administered (placebo (saline solution 0.9%) or PCC (1 ml/kg) according to the assignment specified in the randomization envelope. Treatment is stored in a designated and closed refrigerator and administered in opaque syringes by the same dedicated nurse at a speed of 120 mL/h with syringe pumps.

The investigating team and the patient are blinded (clinician investigator and any MD implied in patient care, nurses, site clinical research assistants). The only unblinded person in the entire process is the dedicated nurse unsealing the envelope and preparing the study drug or placebo to be administered.

Several means are deployed to ensure masking: the dedicated nurse is not involved in the care of the included patient as verified by the monitoring team on every visit. Perfusion material (syringes, filling pipes, and dressings) is opaque, drug storage, randomization, and study drug preparation performed in dedicated protected areas.

The site pharmacy and pharmacy site monitor are not blinded. Statisticians and data monitors are blinded to treatment allocation.

#### Procedure for unblinding if needed {17b}

Unblinding is possible if the information of the allocated treatment is necessary for the care of the included patient. The clinician investigator will retrieve this information from the on-site pharmacist.

To retrieve the information, the referring person will complete on a standard form:

- The contact details of the physician, clinician investigator or not, or the pharmacist who called (name, first name, qualification of the caller, address, telephone number).
- The name of the study.
- The date of the call, the description of the clinical event motivating the call: type of event, start date, and treatment modalities.
- If the event is classified as a serious adverse event.
- The action following this call: unblinding, action to be taken to explore the effect and allow diagnosis, treatment of the effect.

The randomization list is stored in the clinical pharmacy of each participating center. This information will be sent as soon as possible to the study sponsor by e-mail.

The monitoring unit will be systematically unblinded in case of a suspected unexpected serious adverse reaction before reporting to the competent authorities and possibly annually when the annual safety reports are redacted.

### Data collection and management

#### Plans for assessment and collection of outcomes {18a}

An electronic case report form (eCRF, CSonline by clinsight) will be composed for the study. The persons responsible for completing the eCRF will be clearly identified in the task delegation document.

Grenoble University Hospital is the sponsor of the study and is responsible for study oversight and quality controls. Data are collected by the investigating centers’ staff on the electronic case report form. Pseudo-anonymized data are stored by and belong to the sponsor. Participating centers are monitored by a dedicated department, independent of the scientific team and investigators who designed the project. Centers are visited regularly to control patient data, ethical standards, patient safety, respect of masking protocol, and investigational product handling and storage. The primary endpoint is monitored in all patients.

All visits will be the subject of a monitoring report in writing. A copy will be sent to the Principal Investigator.

#### Plans to promote participant retention and complete follow-up {18b}

The primary outcome is collected at day 1 (total blood consumption). This endpoint is easily verified through blood bank data. Patient follow-up will be performed until day 28. Considering the clinical acuity of the patients and their admission to the ICU, no specific action is planned to promote participant retention.

#### Data management {19}

Data management will follow Grenoble-Alpes university hospital procedures (data validation, basic freezing, backup, data reviews before unblinding). A data entry dictionary will be made available to investigators and their teams and traceability of requests for corrections and corrections made on the eCRF.

#### Confidentiality {27}

In accordance with the law (Articles L.1121-3 and R.5121-13 of the Public Health Code), persons with direct access to data will take all necessary precautions to ensure the confidentiality of information relating to experimental drugs, research, and the persons involved, particularly with regard to their identity and the results obtained. These persons, in the same way as the investigators themselves, are subject to professional confidentiality.

During or at the end of the interventional research, all patient data collected and transmitted to the sponsor by the investigators will be pseudo-anonymized. Under no circumstances should the names or addresses of the patients be exposed. Only the first letter of the subject’s surname and first name will be recorded, accompanied by a coded number specific to the research, indicating the order of inclusion of the subjects.

The promoter will ensure each participant’s approval through written informed consent and assure access to their individual data and that no personal data are retained except for the bare minimum required for quality control.

#### Plans for collection, laboratory evaluation, and storage of biological specimens for genetic or molecular analysis in this trial/future use {33}

This is not applicable.

### Statistical methods

#### Statistical methods for primary and secondary outcomes {20a}

The risk of alpha error corresponds to a usual threshold: 5%. All our tests will be bilateral. The initial characteristics of the patients will be represented in a comparative table according to the two treatment groups. Quantitative and qualitative variables will be presented with the usual parameters, mean, and standard deviation as well as numbers and percentages.

##### Analysis of the main outcome

The main outcome is the total blood consumption (RBC, FFP, and platelets). It will be analyzed after verification that the time spent in the study up to H24 is not significantly different between the two groups. If this duration is not different, the total number of units transfused over 24 h between the two groups will be compared using a bilateral student intent to treat *t* test (if the application conditions are not met, we will use a non-parametric Mann-Whitney *U* test). If the duration is different, the amount of blood products used per hour between the two groups will be compared using an intention-to-treat Student *t* test (= total number of bags transfused over 24 h divided by the time spent in research during the first 24 h), in order to control for an attrition bias created by patients who died before 24 h.

The per-protocol analysis will be performed on patients who actually received treatment within 1 h of admission to the hospital.

##### Secondary outcome analysis

We will use the same strategy as for the primary endpoint in order to compare each quantity of RBC, FFP, and platelets, independently; i.e., a Student’s *t* test (if the application conditions are not met, we will use a non-parametric test).

Comparison by a Student’s *t* test (if the application conditions are not met, we will use a nonparametric test) of the values of the Quick Time between the two groups.

The delay between the time of the trauma and clinical hemorrhage control will be compared between the two randomization groups by a Student’s *t* test (if the application conditions are not met, we will use a nonparametric test).

The number of thromboembolic events at D28 will be recoded as a qualitative variable. At least one event (pulmonary embolism or deep vein thrombosis) will be required for this variable. It will then be compared using the chi^2^ test between the two groups (when the conditions for the application of the chi^2^ test are not met, the Fisher’s test will be used).

##### Calculation of the cost of each of the strategies

An average cost of care will be calculated for each group. Micro-costing and gross-costing methods will be used to reconstitute the cost of the hospital stay. Thus, data on the consumption of care will be collected prospectively throughout the entire length of the stay in intensive care (the number and nature of blood products and drug treatments used, additional examinations performed, and the length of stay in each care unit). In this way, we can calculate an average cost of the resuscitation stay in each group. The cost of the overall stay will be reconstituted using the modified GHM method, and it will be based on hospital cost accounting data (calculated within the framework of the ENC: national scale of costs).

Comparison of the cost of the two strategies: The difference in costs between the groups will be tested by a Student’s test or by a non-parametric test (Mann-Whitney tests) in case of non-Gaussian distribution. The choice of the test will be made with regard to the distribution of costs in each group (Shapiro-Wilk test, on raw data or, if necessary, on transformed data). If the distribution of costs is not Gaussian, the construction of the confidence interval of the average cost will be based on the non-parametric bootstrap method.

Deterministic and probabilistic sensitivity analyses will be performed to test the robustness of our estimates. Multivariate analyses will also be implemented to take into account censoring (death and follow-up time) in our cost analyses.

Mortalities at 24 h and 28 days will be compared between the two groups using the Kaplan-Meier model. Length of stay will be compared with a Student’s *t* test (if the application conditions are not met, we will use a non-parametric test).

The GOSE at 28 days will be compared with a Student’s *t* test (if the application conditions are not met, we will use a non-parametric test). The locations of the patient’s hospitalization at 28 days will be compared between the two groups using the chi^2^ test (when the application conditions of the chi^2^ test are not met, Fisher’s test will be used).

#### Interim analyses {21b}

No interim analysis is planned.

#### Methods for additional analyses (e.g., subgroup analyses) {20b}

Since eligibility criteria may select patients without massive bleeding due to imperfect accuracy of the ABC score or wrong clinical judgment, primary and secondary outcomes will also be tested in the population with a massive transfusion (3 RBC within the first post-traumatic hour or at least 10 RBC at day 1).

#### Methods in analysis to handle protocol non-adherence and any statistical methods to handle missing data {20c}

The analysis will be made following an intention-to-treat principle and a per-protocol principle to handle protocol non-adherence. We expect few missing data on the primary outcome since total blood consumption is always recorded and followed by the blood bank in each center.

#### Plans to give access to the full protocol, participant-level data, and statistical code {31c}

Information from the full protocol will be published in a peer-reviewed journal, and the study is registered in https://clinicaltrials.gov. The relevant data analyzed during the development of this study protocol are available upon request from the corresponding author.

### Oversight and monitoring

#### Composition of the coordinating center and trial steering committee {5d}

Coordinating center: Grenoble University Hospital is the sponsor of the study and is responsible for study oversight and quality controls.

Steering committee: Pierre Bouzat, Jean-Stephane David, Jean-Luc Bosson, Bruno Riou, Jacques Duranteau, Jean-François Payen

Data management team: Jean-Luc Bosson, MD, PhD, CHU Grenoble Alpes, Grenoble, France

### Investigational product handling and storage

Shipping of investigational products is under the responsibility of the coordinating clinical trial pharmacy of Grenoble University Hospital. Specifically, they create and ship randomization envelopes, and they order PCC (Kanokad®) to the manufacturer LFB (Les Ullis, France). The biopharmaceutical company LFB is responsible for producing and delivering Kanokad® to investing sites in respect of Good Manufacturing Practices. The coordinating pharmacy in Grenoble also orders and distributes saline solution and opaque devices required for the research to investigating sites. Each site is responsible for storage (2–8°C, away from light), handling and accounting of investigational products received. These elements are verified during monitoring visits.

#### Composition of the data monitoring committee, its role, and reporting structure {21a}

The sponsor appointed a data safety monitoring board (DSMB) that meets every year to analyze safety data (expected and unexpected adverse events). The DSMB is composed of members independent of the research and makes recommendations about protocol termination, modifications, or continuation without modifications.

#### Adverse event reporting and harms {22}

Adverse events are reported blinded to the sponsor by investigating sites and are analyzed by the sponsor safety departments following European regulation on clinical trials on medicinal products.

#### Frequency and plans for auditing trial conduct {23}

An audit initiated at the request of the sponsor or health authorities may be conducted at any time by trained, independent inspectors. The purpose is to ensure the quality of the research, the validity of its results, and compliance with the law and regulations. The auditors/inspectors must have direct access to the source and medical data and to any useful document related to the conduct of the clinical study. The confidentiality of the data and the anonymity of the subjects will be respected.

The investigators agree to comply with the sponsor’s and the competent authority’s requirements for an audit or inspection of the research.

The audit may apply to all stages of the research, from the development of the protocol to the publication of results and the classification of data used or generated in the research.

#### Plans for communicating important protocol amendments to relevant parties (e.g., trial participants, ethical committees) {25}

Any substantial modification, i.e., any modification likely to have a significant impact on the protection of participants, validity and on the results of the research, on the quality and safety of the products tested, on the interpretation of the scientific documents that support the conduct of the research, or on the methods of conducting the research, is subject to written amendment submitted to the sponsor; the latter must obtain, prior to its implementation, a favorable opinion from the ethics committee and an authorization from the national agency for drug safety. All amendments are validated by the sponsor and by all the research stakeholders concerned by the amendment before submission to the ethics committee and the national agency for drug safety. All amendments to the protocol must be made known to all investigators participating in the research. The investigators declare compliance with the protocol. Any amendment that modifies the management of the subjects or the benefits, risks, and constraints of the research is the subject of a new participant information note and a new consent form. The obtainment of informed consent follows the identical procedure as described above.

#### Dissemination plans {31a}

Results will be reported through scientific conferences and peer-reviewed publications following CONSORT recommendations [23]. All participants can be informed of the study results at the end of the trial on request.

## Discussion

The PROCOAG trial explores the management of post-traumatic coagulopathy using pro-thrombin concentrate complex (PCC) in addition to standard blood products and fibrinogen. As opposed to fresh frozen plasma, PCC is readily available at admission and we hypothesized that early correction of coagulation factor deficiency optimizes patient blood management and therefore reduces the total number of blood products transfused within the first 24 h after hospital admission.

Hemorrhage is a leading cause of death in patients with trauma. Although blood products remain the standard for treating hemorrhagic shock, they are a limited and perishable resource. Moreover, concerns are increasing that blood products exert an immunomodulatory effect and negatively affect clinical outcomes [24–26]. Resuscitation strategies that decrease the need for transfusions without increasing complications, therefore, would represent a clinically important innovation.

Eligibility criteria were determined to select patients with severe acute bleeding early in the process so that PCC can be early administrated in the trauma bay on patient arrival. The study hypothesis states an empiric administration of PCC may enhance clotting capacity and decrease total blood product consumption. In consequence, the investigators expect several patients may not require a massive transfusion. The effect of the study protocol on massive transfusion will be investigated in a planned subgroup analysis (see above).

The investigators acknowledge that total blood transfusion is only a surrogate marker of the severity of the bleeding. However, the AVERT Shock trial successfully applied this endpoint based on the rationale that reduction of blood transfusion is an objective of innovative trauma resuscitation [20]. The amount of transfusion has been shown to correlate with severe complications and poor clinical outcomes [10, 21, 27]. Specifically, Moore et al. noted that transfusion of packed red blood cells was the strongest predictor for the development of multi-organ failure (odds ratio = 8.6) [11]. The strategy tested in the present trial hence carries the potential to reduce morbidity, mirrored by the length of stay in ICU and the duration of mechanical ventilation, by reducing the amount of used blood products. The investigators are aware that despite existing guidelines, hemorrhage management may vary. This aspect is acknowledged by the center-based stratification of the randomization process.

Considering the fact that PCC use remains a matter of debate after severe trauma because of an associated potential high thromboembolic risk in this specific patient group; indeed, the use of PCC has repeatedly been shown to result in an excessive overshoot of thrombin generation in both in vitro studies [28, 29] and clinical studies including trauma patients [30]. PROCOAG Trial provides the advantage to explore the safety of empiric PCC administration based on the high clinical probability of active hemorrhage and massive transfusion.

As a pragmatic study, this trial aims at testing the effect of PCC administration in combination with standard care in a *real-life* context. With a high level of proof and quality standard, a positive result might already affect the management of severe trauma patients at risk of massive transfusion as this study investigates PCC efficacy, safety, and cost in this indication.

### Trial status

The trial has obtained all authorizations and recruitment started in December 2017. Enrollment is expected to be complete by June 2021. The protocol version reported here is version 6 as of February 6, 2019.

## Data Availability

Only the principal investigators, the DSMB, and the statisticians will have access to the final data set. There is no plan to share the data, but they are made available for audits and inspections. Anonymized patient data or a full set can be made available on reasonable request. Patients or their relatives are informed that anonymized data can be shared with other groups or used for further studies and have the opportunity to refuse personal data sharing.

## Abbreviations

DSMB: Data safety monitoring board
eCRF: Electronic case report form
FFP: Fresh frozen plasma
GOSE: Glasgow Outcome Scale Extended
ICU: Intensive care unit
NaCl: Normal saline
PCC: Prothrombin complex concentrate
RBC: Red blood cell
